# Shifts in the global migration order and migration transitions in Europe: the cases of Turkey and Russia

**DOI:** 10.1186/s40878-020-00204-2

**Published:** 2020-11-25

**Authors:** Franck Düvell

**Affiliations:** 1 German Centre for Integration and Migration Research (DeZIM), Berlin, Germany; 2 Institute for Migration Research and Intercultural Studies (IMIS), Osnabrück, Germany

**Keywords:** Migration theory, Migration order, Migration transition, Turkey, Russia

## Abstract

This paper takes as a premise that world economics, world politics and global labour are changing and that whilst migration is a driver as well as a consequence of change it is changing, too. For long, conventional research focussed on north-north and south-north migrations, like across the Atlantic or from agricultural and industrialising to industrial countries. This was in part inspired by the economic and political dominance of the ‘global north’, but also driven by a western and Eurocentric bias. Meanwhile, a long period of economic and political transformations and turbulences gave rise to new economic powers, diversified the sending-receiving country matrix and thus fundamentally changed the determinants for international migration. I elaborate the concepts migration order and migration transition to argue that these are useful for analysing the changes in the configuration of sending, receiving and transit states. To illustrate the argument, this article takes Russia and Turkey and developments from the early 2000s as case studies and analyses the shifts in the regional and global migration flows.

## Introduction

This contribution takes as a starting point the emergence of several major migrant destination countries in the vicinity of the European Union during the 1990s and 2010s, notably Russia, Turkey, but also Kazakhstan, Libya and others with China being a further candidate. It challenges the often Western-centric perspective on migration which considers the EU and other western countries as prime destinations and instead acknowledges the changing profiles of countries in global migration processes. In order to analyse the subsequent changes of the configuration of countries, it suggests a rarely applied and usually only colloquially used concept, migration order. First, it sketches the context to this development, notably the interaction between socio-economic transformations and changes in regional and global migration. Second, it elaborates a conceptual framework consisting of migration transition to depict the emergence of countries as new immigration countries and migration order to analyse the structured relations of countries in regional and global migration processes. Third, it analyses and compares changes across some key domains of the migration order which determine the flow of people taking Russia and Turkey, two recently emerging immigration countries, as case studies. Fourth, it concludes that migration transitions of large countries have diversified the flow of people giving rise to a new migration order. This contribution combines the analysis of empirical data with a conceptual elaboration.

## Background: migration in a changing world

The past half century has been characterised by major social transformations and the rise of neo- or liquid modernity (e.g. Alexander 1994). This has been marked by, first, economic crises, like the 1971 oil crisis, the 1987 and 1988 financial crises in Asia and Russia, the East Asian financial crisis of 1997, the crises of 2000 in Argentina and 2001 in Turkey and lately, in 2008, in the US and EU^1^. We have witnessed the economic transformation of the former socialist countries in the ‘global east’, the industrialisation of many countries in the ‘global south’, first the Asian tigers and then rise of the BRICS countries (Brazil, Russia, India, China and South Africa) followed by the MINT countries (Mexico, Indonesia, Nigeria and Turkey) (see O’Neill 2001, 2014). This reduced the number of extremely poor people from 1.9 billion (1981) to 736 million (2015) as well as the number of low-income countries; however, the gap between the poor and the better-off remains wide and deep (World Bank 2013, 2018a).

Second, on the political side there was the fall of the ‘iron curtain’ in 1989, the break-up of the Soviet Union in 1991 and associated rebordering processes in Europe and Eurasia. In particular, ‘the rise of China represents one of the most fundamental shifts in world politics over the past few decades’ (Kastner 2008). Famously, the once bipolar world has been replaced by a multi-polar global political order. These changes are partly driven by revolutions, such as the so-called Arab Spring, and/or wars or civil wars as in Iraq, Afghanistan, Syria, Ukraine, Sudan and elsewhere. These changes not only led to an increase in south-south trade but also to an intensification of south-south and south-east political alliances, as the BRICS council, ASEAN, Mercosur, Eurasian Customs Union, Arab League, Organization of Islamic Cooperation and the African Union.

Third, partly as a consequence, partly as a driver of shifting global orders major demographic changes have been noted. Globally, the population has been growing from 2.5 billion in 1950 in to over 7.6 billion in 2018 and will continue to grow to an estimated 10 billion in 2100. However, this is characterised by significant imbalances: in Europe, the EU, Russia and China populations are largely ageing and partly already shrinking, whereas in the US and Canada populations are still growing which is mostly attributed to immigration. In contrast, in many Asian, Middle Eastern and African countries high birth rates are recorded and populations are still growing; notably India has become a population giant and will soon have more workers than China (Gökay 2009). Another important feature is the gender imbalance and the relative shrinking of the female populations in China and India. Thus, demographic frontiers emerge between regions and countries with ageing and shrinking and countries and regions with young and growing populations.

Fourth and finally, also migration politics such as labour recruitment schemes or migration restrictions, visa requirements respectively visa free travel arrangements, the types of rights offered or refused to migrants, the public attitudes towards immigrants and the migrants’ perceptions of the countries of origin and destination all contribute to driving (future) migrations (e.g. Timmerman et al. 2014).

These developments change and shape the movements of people. Currently, international mobility has reached unprecedented levels: air travel has grown by 700% in 10 years; international tourist arrivals have increased by 100% over the last 20 years and has reached 1.7 billion international arrivals (UNWTO, various; ATAG (Air Transport Action Group) 2018); international migration has increased from 154 million in 1990 to at least 258 million (United Nations 2017) whilst by 2016 another 710 million people have expressed an ambition to migrate (Gallup 2017). Nevertheless, UNWTO finds that ‘the large majority of international travel takes places within traveller’s own region, with about four out of five worldwide arrivals originating from the same region’ (UN World Tourism Organization (UNWTO) 2013, p. 10).

So far, in many regions and countries populations have been un-mixing (Van Hear 2012) along national, ethnic and religious fault lines which led to significant migrations, as in the former Soviet Union or former Yugoslavia as well as in Iraq or Nigeria or in the case of Christians fleeing Muslim countries. In 2017, no less than 49 state-based conflicts and 82 non-state conflicts (PRIO 2017) generated over 25.4 million refugees and 40 million internally displaced persons, mostly in the ‘global south’. Some declining economies have become major sending countries, such as Ukraine, Romania and Moldavia; some previous receiving countries have been hit hard by the 2008 crisis and turned into migrants sending countries, such as Portugal, Greece and Spain, other emerging economies go through a migration transition from migrant sending to receiving countries, such as Turkey, or have become attractive destinations, as, for instance, South Africa. Also, Russia has become an important destination country, no longer only for citizens from former Soviet countries but increasingly also for international migrants. North-North migration represents 22% of all migration, South-North migration around 40%, South-South migration (including movements to CIS countries) consists of more than a third of all migration whilst another 5 % is North-South migration (IOM 2013). However, I dispute this categorisation and instead suggest introducing ‘East’ as a separate category; at least 5–10% of all migration is East-East migration and some percentage is South-East migration. The constantly changing configuration of states and their function as net sending or receiving units I conceptualise as global migration order and the changes as migration transitions as elaborated in the following section.

### Theoretical framework

Migration order is a relatively new and rarely used concept. It was first applied by Van Hear (1998), as it seems, to describe changing flows mainly of refugees. He links micro-level social processes to macro-level structures within which these occur. In his subsequent work, he adds features such as shifts in the global economic order, the rise of new economic powers in the global South and East as well as the consequences of restrictive migration regimes in the global North to explain shifting migration flows (Van Hear 2012). However, he does only sketch the contours of the concept migration order and somewhat nebulously refers to its ‘features’ and ‘domains’. Van Hear (1998) specifically considers crises as periods of chaotic events ─ similar to the crises sketched above ─ which facilitate the investigation of changes in migration orders; in contrast, most literature on social, political or economic orders rather takes a longitudinal approach. Pieke (2007) taking the case of China too used the concepts ‘world migration order’ and ‘hubs in the global migration order’ to analyse changes in global economic and political opportunity structures in the sending and receiving contexts, new geographies and new flows of new types of migrants. However, neither concept he elaborates further. Finally, Strangio and De Rose (2015, p. 228) suggest ‘we might see new patterns of migration, new sending and receiving countries and the rise of a new migration order’. Neither of these authors further substantiate this concept or embeds the notion of a migration order in broader theories on social, political or economic orders; instead, so far, the concept has rather been used in a colloquially, improvised and rather descriptive than analytical fashion. Therefore, some further elaboration is required.

In general, the search for order or structure implies the search for meaning and explanations of micro-level individual behaviour and macro-level social processes. The concept of a ‘social order’, for example, typically refers to macro-level social forces including social institutions, notably family, religion, education, media, law, politics, and economy, and patterns of institutionalised relationships. It implies some structured, regular, logic and patterned structure, behaviour and interaction between individuals, collectives or organisations including states and consequently some predictability and thus complex interactions between macro-level structures and individual behaviour (e.g. Frank 1944; Hayek 1948). ‘Order’ does not imply, however, that macro-level forces simply determine individual behaviour, as Thorlindsson and Bernburg (2004) argue referring to Durkheim. Merton too rather (Merton 1957) understands social orders as opportunity-constraints structures within which individuals act, hence also migrants, as in our case. Giddens’ (1984) structuration theory and his analysis of the interplay of structure and agency further reinforces this point. These theories on orders are thus helpful to understand the macro-level factors underlying individual behaviour by migrants (also see Bakewell 2010). In political science the concept of an order is utilised to analyse the relation of states as a ‘world order’ and, where applicable, a ‘system of global governance’ (Slaughter 2004, p. 15). Meanwhile, Cox (1987) describes ‘world order’ as the structure of states understood as a terrain of conflicts between individuals, civil society, state and global actors, this notion in particular facilitates the analysis of the impact of migration policies on migration flows. The notion of a migration order is thus particularly useful to analyse the relations of states in migration processes. Similarly, the literature on ‘international economic order’ (e.g. Gilpin and Gilpin 2001) analyses the different roles and interactions of (more or less developed) states in international economic matters and specifically looks at processes of economic transitions. Since migration is closely related to economic disparities the notion of an economic order almost calls for being extended to migration matters. Finally, this scholarship emphasises that the world order is in a constant state of change (e.g. Cox 1987). These epistemic practices inspire me to also apply the concept of ‘order’ to conceptualise the configuration of states in migration processes.

The concept of a migration order is closely related to the concept of migration systems. But these are rather ‘spatial processes with a clear geographic form and structure’ (Massey et al. 2008, p. 60). Conventionally, a migration system is thought of as a pair or small group of countries, usually one or more sending and a receiving country (‘countries that exchange relatively large numbers of people … [and] concomitant flows of goods, capital, ideas and information’, resulting in ‘economic, political and cultural links’ forming ‘a network’ representing ‘a migration system’ (ibid.). However, Giddens (1979) emphasises that orders or structures are different from systems in that the latter usually connote the working together of parts of a mechanism and thus a common *purpose* whereas there is no common purpose in the orders referred to here. Therefore, the concept of a migration order is more adequate than migration systems to depict the configuration of states not primarily brought about by intent and for a purpose but as the consequence of the collective human agency of the individuals who migrate. Also, because of the spatial restriction of migration systems, I suggest to conceptualise the sum of links between sending and receiving countries or small-scale migration systems as a migration order which can thus be understood as the overarching structured configuration of the sum of sending and receiving countries of migration.

The concept of a migration order, just like the economic or world order, does not imply static conditions; instead, all readings acknowledge their dynamics. In migration theory, the concept of migration transition has long been applied to analyse the changing characteristics of cities or states in migration processes. It was introduced by Zelinsky (1971) and links different stages of modernisation (pre-modern, early transitional, late transitional, advanced to super-advanced societies) to different levels and types of migration (circular, rural-urban, urban-urban, international). Often, the concept refers to a country’s shift in particular from a net emigration to a net immigration country (e.g. Fields 1994). It thus resonates well with Cox’ (1987) historicist understanding of the world order. However, the original concept of migration transition has been strongly criticised and should thus not be understood, as initially suggested by Zelinsky, as a determinism or path-dependent mechanism (see Skeldon 2012, de Haas 2010). In contemporary reading the concept does not imply that all countries move from one stage to the other, i.e. from a lower to a higher stage, or suggest that countries will remain on a certain stage. Instead it suggests that countries can remain on the sending stage or, once they have converted to the receiving stage, can fall back into the sending stage. Migration transition theory has been applied to analyse changes in migration that are related to Europeanisation and globalisation (Findlay et al. 1998, Skeldon 2012) and is useful to also depict such changes in other parts of the world. Skeldon (2012) maintains that migration transition theory is nevertheless useful to linking migration systems to wider socio-economic change and I take it as given that this also applies to the migration order.

For the purpose of defining migration order I thus take from Merton the notion of social structure, apply this to migrants and basically rephrase his definition of social order as the ‘structure of states [with regards to migration], the behaviour of man with that and the consequences of that behaviour’ (1968: 71)^2^. Referring to Giddens’ (1984) structuration theory I further argue that a migration order is the result of a circular process of structuration in which the actions of agents – policy makers, entrepreneurs, and migrants – are shaped by and shape the structure. I utilise Cox’ notion of the world order to better understand the interplay of sending, receiving and/or transit states of migrants and add to this Slaughter’s notion of world order as governance structures ─ notably with regards to migration management ─ applied to the field of migration. The concept of a migration order complements the demographic, economic and political orders and thus refers to the configuration of macro-, meso- and micro-level factors and facilitates analysing structured, regular, logic and patterned behaviour and interaction between individuals, collectives or organisations with regards to migration processes. To the concept of a migration order I integrate the concept of migration transition to facilitate analysing changes in the position of states in the migration order.

On these grounds I operationalise Van Hear’s (1998) analysis of migration orders by looking at three key domains: macro-level economic and demographic (e.g. GDP per capita, GNI per capita, HDI, population growth and ageing, unemployment rates and remittances) as well as political determinants (e.g. visa and migration policy, regional and global migration regimes, IR), the meso-level infrastructural determinants (e.g. travel networks) and finally some micro-level determinants (e.g. migration aspirations and individual characteristics partly reflected by the HDI and the Happiness Index). This is not yet exhaustive as a comprehensive analysis would need to entail migration networks as another meso-level factor.

### Case studies

In the following, this paper analyses and compares the rise of the Russian Federation (short Russia) and Turkey as economic powers and their key demographic, economic and political domains, their transition into major immigration countries, the rise of secondary destination countries in the regions, notably Kazakhstan and thus the emergence of a new migration order in Eurasia, Eastern Europe and the Middle East.

The two cases are similar in that from the early 2000s both have enjoyed significant economic growth, aspire to consolidate their roles as regional economic powers, have introduced relatively liberal visa regimes and thus become net immigrants receiving countries. Both countries also display imbalanced economies, that of Russia relies heavily on oil and gas revenues whereas Turkey’s economy is based on a bubble fuelled by cheap money (Forbes 2019). The cases differ in that Russia has an ageing and shrinking population whereas Turkey’s population is still growing though also already began ageing. And whilst Russia is the centre of a political bloc Turkey has for long been allied with the Western bloc. Finally, Turkey has high levels of internal geographic mobility (Güngördü 2019) whereas in Russia mobility is low (Khramova 2012). All of these factors shape in different ways the movements of people.

The data used in this article mostly cover the period 1998 to 2012. This is because the subsequent period is characterised by exceptional events which distort previous trends; also, changes in migration law render comparison of older and newer data impossible. But because the purpose of the article is not to analyse the latest situation in the two countries but to substantiate a conceptual proposition this shall be adequate.

### The migration transition of Russia

Migration within the Soviet Union and Russia has for long been neglected by international migration research. For instance, in the earlier versions of Castles’ and Miller’s famous ‘Age of Migration’ compendium a map of major international migrations flows and systems omitted flows in the former Soviet Union. Only slowly is the post-Soviet migration space being acknowledged by international migration research.

In 1995, the flow of people to Russia only consisted of 10.3 million arrivals; by 2001, this more than doubled to 21.6 million and further increased to 25.7 million in 2012 (UN World Tourism Organization (UNWTO) 2012, also see Index Mundi 2012). However, numbers have been fluctuating annually whilst the overall increase has slowed down significantly. Kazakhstan in 2012 also recorded 4.4 million arrivals (UN World Tourism Organization (UNWTO) 2012, UN World Tourism Organization (UNWTO) 2013). In the meantime, international travel of Russians has doubled too, from 21.3 million in 1995 to 39.3 million in 2010 (Stark Tourism 2013). Finland, an important entry point to other Schengen-zone countries, Turkey and China with 3.1 million arrivals each were the prime destination countries followed by Egypt (1.6 million), Estonia (1.5 million) and Germany (0.9 million) as main non-CIS destinations. By 2014, Russia had become the 5th largest sending country of global travel (UN World Tourism Organization (UNWTO) 2014). A small proportion of these international travellers have been migrants. However, how volatile this is has recently become apparent when Russia and Ukraine fell out and Ukrainian migration to the East diminished whilst migration to the EU increased, partly facilitated by a new visa liberalisation.

**Fig. 1:**
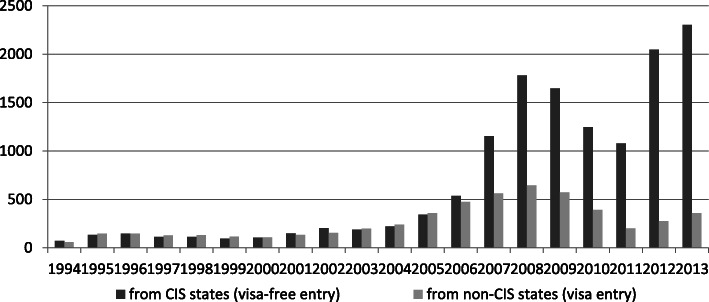
Annual foreign labour inflow to Russia, 1994–2013, thds. (work permits, since 2011 incl. Labour licences) (for the figure see Ivakhnyuk (2014))

In 1994, just after the end of communism, the stock of labour immigrants in Russia was around 120,000, with similar numbers coming from other former Soviet republics, the Commonwealth of Independent States (CIS) countries, and non-CIS countries. Within the next 10 years, this slowly increased to 384,000 but from 2004, labour migration rose sharply to 2.42 million in 2008 and, after some decrease during the years of the 2008–11 economic crisis, jumped up again to 2.66 million in 2013. During this period, annual regular net immigration has oscillated around the + 300,000 margin. These numbers, however, only reflect regular migration and since ‘up to 70 percent of labour migration within the Eurasian migration system is irregular/unregistered migration’ (Ivakhnyuk 2014, p. 60) there could have been up to another 6.5 million irregular labour migrants in Russia, bringing the total up to 9 million, though other sources refer to up to 11 million (Lyubinskaya 2013). Hence, around seven to 10 % or even more of Russia’s labour force consists of immigrant workers (Fig. 1).

During the period 1994 to 2008, migration from CIS and non-CIS countries increased at different paces; whilst labour migration from non-CIS countries tripled labour migration from the CIS even eightfold (IOM 2009; also see ICMPD 2006). Initially, most migration consisted of ethnic Russians returning from the diverse former Soviet republics and began to ethnically diversify. From 1994, new large-scale migration emerged with Russia as the main and Kazakhstan as a secondary destination country and Armenia, Azerbaijan, Georgia, Kazakhstan, Moldova, Tajikistan, Turkmenistan, Kirgizstan, Ukraine and Uzbekistan as main sending countries. In 2008, the largest absolute numbers of labour migrants were from Uzbekistan (642,700), Tajikistan (391,400) and Ukraine (245,300). This trend continued into the 2010s. In addition, in 2008, 281,700 labour migrants from China were recorded. Whilst Uzbekistan remains the major labour supplier in the region China sent the third-largest number of registered migrants to Russia; Turkish regular labour migrants represent two to 4 % of regular labour migrants. It is noteworthy that most migration has been low-skilled and that higher skilled migrants, notably from Ukraine rather migrate west.

In addition, Russia has also been receiving international refugees albeit numbers are normally small. For instance, in 2014, UNHCR (2014a, 2014b) had registered 3458 refugees and 1240 asylum seekers. However, by June 2014 another around 110,000 displaced persons from Ukraine where assumed to be in Russia though some sources report much higher numbers (United Nations 2014).

Above description illustrates that by 2010 Russia had become a net immigration country and had thus gone through a migration transition. The next section considers the determinants across three key domains to be considered for analysing the migration order.

### Determinants of the migration transition of Russia

Migration in Eurasian is driven by enormous discrepancies in the countries’ GDPs and demographic characteristics and facilitated by a common Soviet history, a liberal immigration policy meaning visa-free travel, geographic proximity, a common travel system and some related political, linguistic and cultural similarities ─ Russian is the Lingua Franca of the region.

### Demography

Seven countries in the regions have increasing populations (Armenia, Azerbaijan, Kazakhstan, Kirgizstan, Tajikistan, Turkmenistan and Uzbekistan) whereas five countries have shrinking populations (Russia, Ukraine, Belarus, Georgia and Moldova). Two of the economic and industrial heartlands, Russia and Ukraine, expect significant population ageing and decreasing whereas Kazakhstan, the other economic power, still enjoys population growth. These population discrepancies have been an important driver of migration (United Nations, Department of Economic and Social Affairs (UNDESA), Population Division 2019) and thus a contributor to the changing migration order (Table 1).

**Table 1:** Population size and growth, by country, thousands

Country	2010	2025	Increase (+) / decrease (−)	Total fertility rate (2013)
Russia	143,618	136,967	- 6651	1.6
Uzbekistan	27,769	32,991	+ 5222	1.8
Kazakhstan	15,921	18,116	+ 2195	2.3
Azerbaijan	9095	10,309	+ 1214	1.9
Tajikistan	7627	10,539	+ 2912	2.8
Kyrgyzstan	5334	6557	+ 1223	2.7
Turkmenistan	5042	5951	+ 909	2.1
Armenia	2963	2989	+ 26	1.6
Ukraine	46,050	41,560	- 4490	1.3
Belarus	9491	8773	- 718	1.5
Georgia	4389	4080	−309	1.8
Moldova	3573	3206	- 376	1.6

Sources: United Nations Population Prospects, http://esa.un.org/unpd/wpp/unpp/; CIA database, https://www.cia.gov/library/publications/the-world-factbook/fields/2127.html

### Economy

**Fig. 2:**
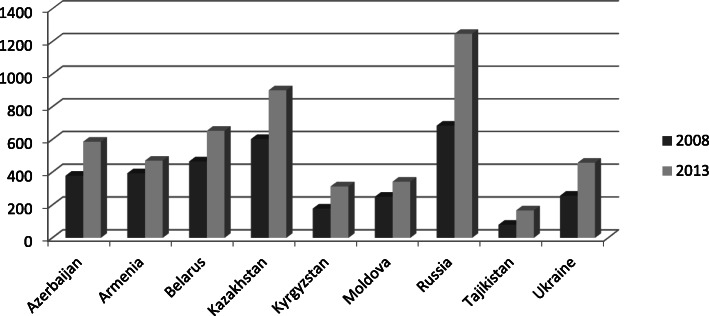
Average monthly wages in the Eurasian migration system countries, 2008 and 2013, USD. Source: Data of the CIS Interstate Statistical Committee: www.cisstat.com

From 2000 to 2010 Russia’s GDP six-fold from 400 billion to 2.3 trillion. Her GDP per capita and the GNI per capita have been significantly higher than those of all other eastern and southern countries in the region (see Table 2). Also, average wages differ significantly and are highest in Russia, second highest in Kazakhstan and lowest in Tajikistan, Kirgizstan, Moldova and Ukraine. But whilst in 2008, Russian and Kazakh wages were similar by 2013 Russian wages almost doubled widening the wage gap between Russia and other CIS countries. Wages were three times higher than in Ukraine and seven times higher than in Tajikistan (Fig. 2). Russia’s economic might put her centre stage on the economic order and determines a migration order in which Russia is the key destination country of migration. As a consequence, a system of enormous interdependence has emerged in Eurasia in which Russia depends heavily on immigrant labour whereas several sending countries heavily depend on remittances (Table 3).

**Table 2:** GDP and GNI per capita in $ and HDI, Russia compared with its neighbours and some relevant sending countries (2012) (World Bank 2018b and 2018c, UNDP 2017)

Country	GDP per capita	Difference with Russia in %	GNI per capita	Difference with Russia in %	HDI/rank
Russia	14,612	100	13,860	100	0.778 (54)
Kazakhstan	11,935	86	11,380	82.1	0.757 (70)
Turkey	10,666	76.2	10,950	79	0.759 (69)
Turkmenistan	7987	57	6880	49.6	0.698 (103)
Azerbaijan	7392	52.8	6030	43.5	0.747 (75)
China	6807	46.6	6560	47.3	0.719 (91)
Ukraine	3867	27.6	3960	28.6	0.734 (83)
Georgia	3508	25	3570	25.8	0.744 (81)
Armenia	3338	23.8	3790	27.3	0.730 (87)
Moldova	2038	14.6	2460	17.7	0.663 (114)
Uzbekistan	1878	13.4	1900	13.7	0.661 (116)
Kirgizstan	1263	9	1200	8.7	0.628 (125)
Tajikistan	1037	7.4	990	7.1	0.607 (133)

**Table 3:** The role of migrant remittances for specific CIS countries, 2013

	million USD	% of GDP
CIS countries	24,786	
Tajikistan	3927	51.9
Kyrgyzstan	2113	31.
Moldova	2248	24.6
Uzbekistan	7878	16.3

Source: Bank Rossii (2014)

### Policy and travel infrastructure

These migrations are shaped by the Soviet past and facilitated by Russia’s contemporary foreign, immigration and transport policy. Generally, Russia aims to re-invent herself as a world power, including a new emphasis on Russia’s relations with Eurasia as well as with other BRICS countries (Monaghan 2013) which all shape its migration policy. Further to this, through forced migration Russia is affected by troubles in its neighbourhood, notably the Caucasus, Central Asia and recently Ukraine but less by the troubles in the Middle East.

Russia applies a mixed and complex system of visa-free entry under varying conditions^3^ for CIS^4^, some Central- and South-American and few other countries^5^ and visa requirements for most other non-CIS countries (Russian National Tourist Office 2014). Her labour market access Russia regulates by a mixed system of quota and non-quota categories. Its conventional quota system, however, is inconsistent with the newly introduced non-quota labour permit system and brings about high levels of irregularity (Ivakhnyuk 2014).

Russian Railways (2019) claim to be ‘the strongest link in Eurasia’, its website claims they are ‘a strategic player expanding East-West and North-South Eurasian transport corridors and integrate Russia into the global economy’. Every year, they transport more than one billion passengers. But while ‘the number of airline flights between Russia and the capitals of Central Asia doubled in the 1990s’, flights and trains remained ‘expensive for the majority of migrants’ so that most have been ‘compelled to use buses’ (Laruelle 2007).

### Implications

**Fig. 3:**
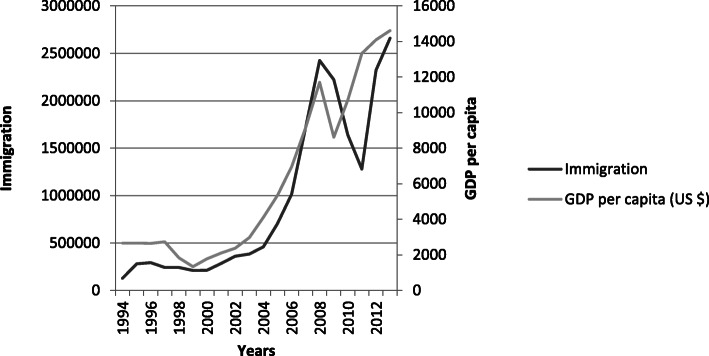
Development of GDP per capita (in hundred) and labour immigration in Russia, 1994–2013

Above description reveals a striking coincidence between the development of Russia’s GDP per capita and the level of immigration to Russia, the rise in immigration almost parallels the rise of the GDP, though with some time lag (Fig. 3). This implies that Russia’s increasing economic power combined with its decreasing population and lax migration regulations trigger and facilitate migration from the comparably weak neighbouring economies with their growing populations. These coincidences explain the migration transition of Russia into a major immigration country and its new powerful position in the migration order. In 2010, in the CIS countries it was found that indeed ‘Russia [is the] No. 1 desired destination for permanent migration, temporary work’, 55% of the working age people in these countries desire to migrate to Russia (Gallup 2010a).

### The migration transition of Turkey

Turkey has conventionally been known as a major sending country of labour migrants to some EU countries. From the 1990s, mostly irregular transit migration to the EU has triggered increasing research interests; in particular the large-scale influx of Syrian refugees attracted fresh research on Turkey. However, matters are more intricate than this and from the late twentieth century the country has slowly been turning into an immigration country^6^.

It is estimated that from the early 1920s to late 1990s, primarily ethnic Turkish Muslims mostly from the Balkan and various parts of the former Soviet Union migrated to Turkey consisting of around 1.7 million people (Içduygu and Biehl 2012; Kirişçi 2003). Additionally, from the 1980s to 1990s, an average of 45,000 ethnic Turks annually returned from Germany dropping to around 35,000 during the 2000s (Pusch and Splitt 2013); the majority of the more recent returnees have been of working age (Baykara-Krumme and Nauck 2011). In contrast, the recent period is characterized by increasing numbers of non-ethnic Turkish and/or non-Muslim travellers and immigrants arriving in Turkey for various purposes such as business, employment, education, recreation and retirement or international protection.

The total flow of foreigners to and from Turkey has almost tripled from 23 million in 2001 to around 63 million in 2011 arrivals and departures; in addition, 23 million journeys of Turkish citizens were recorded in 2011. Mobility to and from Turkey increased across all countries; however, several regions and countries stood out. Flows from Germany were highest and stood at 9.5 million movements; 5 million British citizens represented the second largest group from the EU followed by 2.4 million from the Netherlands. Travel to and from the CIS quadrupled to almost 13 million visitors, also 5 million from Asian countries, 4 million from the Middle East and Gulf countries, 1.5 million from the USA and 0.9 million from African countries were recorded. Notably flows from Russia have almost quintupled from just 1.5 million in 2001 to almost 7 million in 2011 making Russia the second most important country of departure and arrival. Finally, flows from China quadrupled, though remained low at 192,000 (Turkish Statistics Institute (Turkstat) 2011). These numbers illustrate that Turkey is well integrated into global mobilities.

The World Bank (2017) estimates that during the period 2009 to 2013 net immigration was positive amounting to 350,000 individuals, up from minus 50,000 in 2007. Also, some forms of short-term visits on tourist visa were rather disguised forms of transnational migration: tourist visa holder from the EU have often been de facto retirement migrants (International Strategic Research Organization / Uluslararası Stratejik Arastirmalar Kurumu (ISRO/USAK) 2008, p. 6), other short-term visits from CIS countries as well as Africa have actually often been repeated entrants for economic purposes, either suitcase trade or short-term or seasonal employment which subsequently led to longer stays. In addition, a specific flow consists of migrants who enter Turkey with the intention of moving on to the EU; but these so-called transit migrants often stay for considerable periods of time, either on a visa, whilst applying for asylum or irregularly and have de facto been immigrants.

On the other hand, by the early 2000s fewer than 50,000 Turkish nationals went to the European Union, one third were family members, the others students or workers. In 2011 still only 53,800 usually temporary labour migrants were officially recorded (Içduygu et al. 2013). From these numbers it can be calculated that at least 175,000 people annually entered Turkey to stay for longer periods of time, outnumbered emigration and turned Turkey’s migration balance positive. Other sources claim that even up to ‘250,000 people ...enter Turkey each year with the intention of staying longer, be it for education, employment, or retirement’ (Evin et al. 2010).

In 2000, the stock of foreign-born persons in Turkey reached 1,278,671 (State Institute of Statistics (SIS) 2003); most of these were ethnic Turks, two thirds born in Bulgaria, Germany, Greece, Macedonia and Romania. By 2010, this rose only marginally to around 1.4 million individuals (World Bank 2010). Many of these had acquired Turkish nationality, ─ from 1997 to 2009 355,865 persons were naturalised (Içduygu and Aksel 2012). Remarkably, only a minority of these seemed to have been ethnic Turkish and/or Muslim and a majority have been of non-Turkish and/or Muslim background (Içduygu and Aksel 2012).

In contrast, in 2000, only 234,111 persons were recorded as immigrants by the Turkish Statistical Institute (Turkstat) (2007). From, 2001–2005, the number of residence permits issued was around 160,000 annually and then increased to around 180,000 from 2006 to 2010; of these an average of 20,000 were issued for the purpose of employment, another 30,000 for studying whilst a further around 10,000 work permits were issued by the Ministry of Labour and Social Security (Içduygu and Aksel 2012). By 2011, there were around 220,000 foreign holders of residence permits in Turkey OECD (2013) originating from 176 different countries (OECD 2012); the top nationalities were Bulgarians, Azeris, Iranians, Iraqis, Russians, Germans, US Americans, Former Yugoslavians, Afghans, Kazakhs and Greeks.

In addition, Turkey has received increasing numbers of asylum seekers and displaced persons from many parts of the world. Hence, to the above figures 32.906 international refugees must be added (2013) (UNHCR 2013a, 2013b), 12,982 from Iraq, 8507 from Afghanistan, 6683 from Iran and 2049 from Somali; from 1997 to 2011, 101,067 people had applied for asylum (Içduygu and Aksel 2012). There were also 764,820 registered displaced persons from Syria (April 2014) (UNHCR 2014b) plus another few hundred thousand non-registered Syrians. For various reasons these have initially been mostly transient populations, though from 2016 refugees began staying in larger numbers. Finally, there are also significant levels of irregular immigrants in Turkey, from 400,000 to well under one-million (Düvell et al. 2015) (Table 4).

**Table 4:** Stock of international immigrants in Turkey by country or region, 2005–2012^b^

Country/Region	Estimates/Year
Bulgaria	51,787 (2008) (International Strategic Research Organization / Uluslararası Stratejik Arastirmalar Kurumu (ISRO/USAK) 2008)
Greece	6191–62,463 (Içduygu and Sert 2009, IOM 2008)
div. African countries	50000^a^
Macedonia	33,242 (2005)^a^
Germany	20,000–120,000
Russia	30,000–100,000^a^
Afghanistan	< 30,000, incl. 9000 refugees^a^
Ukrainian	20,000 (MFA 2012)
Armenia	10–20,000, 72,000 (2002)^a^
Iran	13,667 (2005) (IOM 2008), 8624 (only refugees in 2010) (OMID 2010)
Azerbaijan	10,879 (2005) (Içduygu and Sert 2009)
UK	7940 (ISRO/USAK 2008)
Georgia	6868 (2005) (IOM 2008), 13000^a^ (2010)
Iraq	5927 (permits, 2006) (Içduygu and Sert 2009)
Philippines	5000 (Independent Balkan News Agency (IBNA) 2013)
Moldova	4674 (2006) (Içduygu and Sert 2009); > 8600 (2008)
Total (lowest/highest est.)	262,132 / 506,578

^a^Own estimate based on various sources quoted in this article

^b^Compiled from the sources quoted in this article or given in the table; numbers are not comparable as they were collected or estimated in different years

According to the various figures discussed in this article, in 2012, the proportion of immigrants of the total population of 75 million was 1.4 million, or 1.93% if taking the foreign-born persons stated by the World Bank (2010), 2.5% if taking UN figures (United Nations 2013), 2.1–2.4 million or 2.8–3.2% if adding to this refugees including Syrians or 2.5 to under 3 million or 3.3–4% if also adding the estimated irregular immigrant population. This is confirmed by the 2011 Population and Housing Census finding that 100,000 of 3.2 million surveyed households, 3.15%, identified themselves as immigrants meaning that they were residing abroad one year ago (TurkStat 2013).

This description illustrates that by 2010 Turkey too had become a net immigration country and had thus gone through a profound migration transition. The next section considers the determinants across three key domains of the migration order.

### Determinants of the migration transition of Turkey

Turkey stands out in a volatile region as a prosperous, relatively stable and still even comparably liberal country. It shares with its neighbours and other countries in the region some common history, culture and religion.

### Demography

In 2012, Turkey had a population of 75 million that is expected to increase to 90 million though it is already slowly ageing. There is a discrepancy between the youthful east and the ageing west triggering some internal migration. But as most of the countries in the region, except in the EU and in some CIS countries display similar demographic characteristics these do not account for drivers of migration.

### Economy

Of the 16 countries in the region respectively the countries where migrants to Turkey originate from only three had higher GDPs and GNIs per capita than Turkey, Greece, Russia and Kazakhstan (see Table 5, in grey colour) whereas all others have sometimes significantly lower GDPs and GNIs per capita.

**Table 5:** GDP and GNI per capita in $ and HDI, Turkey compared with its neighbours and some relevant sending countries (2012) (World Bank 2018d, UNDP 2017)

Country	GDP per capita	Difference with Turkey in %	GNI per capita	Difference with Turkey in %	HDI
Greece	22,083	220	25,460	254	0,86
Russia	14,037	140	12,700	127	0.788
Kazakhstan	11,935	120	9750	97	0.754
Turkey	10,666	100	10,830	100	0.722
Azerbaijan	7392	74	6030	60	0.734
Bulgaria	6986	70	15,390	153	0,782
Iran	6815 (2011)	68	4290 (2009)	43	0.742
Iraq	6455	65	5870	59	0.59
Ukraine	3867	39	3500	35	0.74
Georgia	3508	35	3280	32	0.745
Armenia	3338	33	3720	37	0.729
Syria	3289	33	2610 (2010)	26	0.648
Morocco	2902	29	2950	30	0.591
Moldova	2038	20	2250	22	0.66
Pakistan	1290	13	1260	13	0.515
Senegal	1032	10	1040	10	0.47
Afghanistan	687	0.6	570	0.6	0.175

There were also significant discrepancies in the economic participation rate; it was lower in eight other countries in the region than in Turkey. The total unemployment rates as well as youth unemployment rates between Turkey and its neighbours and other sending countries differed significantly, too; eight of 17 countries had higher unemployment levels and nine had higher youth unemployment rates than Turkey (Table 6).

**Table 6:** Unemployment and participation rate (figures for last available year) (ILO 2012; United Nations 2012)

Country	Participation rate	Unemployment in %	Youth not employed or in education in %
FYR Macedonia	55.5	31	55.3
Greece	42.2 (2001)	24.2	44.4
Armenia	63	17.3	45.5
Iraq	41.3	15.3 (2008)	n.d.
Georgia	65.2	15	35.6
Iran	38.3	13.5	26
Bulgaria	52.5	12.3	26.6
Senegal	55.2	10.4	n.d.
Morocco	49.2	9	17.9
Turkey	49.4	9.2	17.5
Syria	44.3 (2008)	8.6 (2010)	19.2 (2010)
Afghanistan		8.5	n.d.
Ukraine	48.3 (2007)	7.5	18.6
Azerbaijan	64.4	5.9 (2011)	14.7
Russia	67.7	5.6	15.5
Moldova	40.7	5.6	14.9
Pakistan	45.8 (2010)	5.5 (2009)	7.7
Kazakhstan	71.2	5.3	3.8

These figures, however, are of limited use as they veil other important factors; notably, they do not reflect people who were recorded unemployed whilst nevertheless working irregularly, a pattern which is particular widespread in Turkey. Also, simple employment rates do not account for the working poor; for example, in Ukraine, Armenia and Georgia over 15% of the workers are classified as working poor. Other people in employment might still be working below the level of their education and thus cannot realise revenues according to their skills. Hence, even if the discrepancy between employment opportunities is low, as between Turkey and Ukraine, jobs in Turkey might still be more attractive because they are of higher status or because salaries are higher. Thus, unemployment levels are an imperfect indicator.

### Policies and infrastructures

Turkey is embedded in a politically extremely volatile region. There is continuous violence in Syria, Iraq, Afghanistan and Palestine, tensions between Russia and Ukraine and frozen conflicts in Georgia, Armenia and Azerbaijan, Moldavia and Cyprus. Relevant for the purpose of this paper is that these troubles generate short-term and long-term forced migration (refugees) who seek shelter in the safe countries in the region of which Turkey is the main recipient.

Further to this, Turkey has introduced a rather liberal though complex visa regime with many countries in the region and beyond (Acikgöz and Ariner 2014). Turkey has permitted visa-free entry to citizens from 55 countries or has been issuing almost unconditional e-visa^7^, also citizens from a visa list country who already hold another OECD country’s visa have been exempted from Turkish visa requirements or could easily obtain an e-visa (Ministry for Foreign Affair 2014). In 2013, e-visas have replaced the previous sticker-visas that were simply issued on the border for a small fee. This policy is driven by the aim to (a) boost the economy and notably tourism and (b) expand and strengthen Turkey’s international relations through mobility and migration.

It seems, that apart from neighbouring Syria, Iraq, Iran and Bulgaria most international travellers to Turkey arrive by air. Notably Turkish airlines have expanded their businesses so that a vast network serves many countries in Europe, Eurasia, Asia and Africa (New York Times 2012). This linking of places and transportation of people is part of Turkey’s ‘geopolitical aspirations’ and globalisation strategy (Anaz and Akman 2017) and provides a crucial infrastructure for mobility and migration.

### Implications

**Fig. 4:**
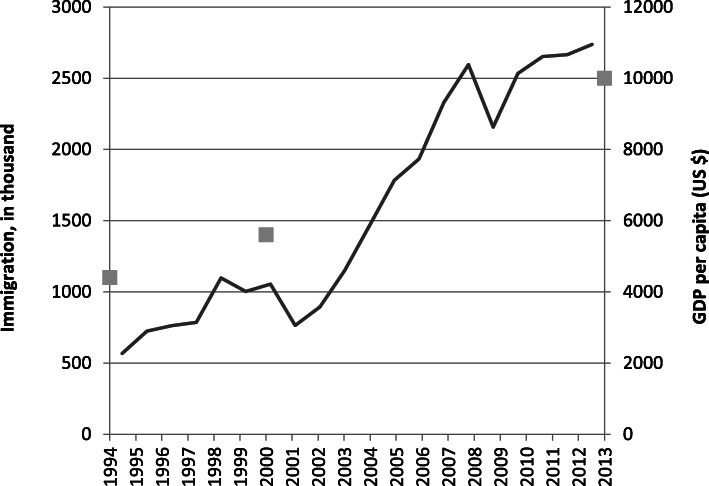
Development of GDP per capita and immigration to Turkey, 1994–2013

Section 3.2. demonstrates that from around 2010 Turkey’s net migration has turned positive. The fact that there were permit holders from 176 countries implies a hugely diverse immigrant population, suggests that there were immigrants from almost any country in the world and demonstrates that Turkey is well integrated into global migration processes. In 2009, 1.9 million people expressed the desire to move to Turkey permanently, mostly from Russia, Iran, Azerbaijan, Germany and Syria (Gallup 2010b). This typifies a migration transition driven mainly by her economic growth (see Fig. 4) and facilitated by a liberal visa regime which has turned the country into a major migrants-receiving country within the global migration order.

## Conclusion

**Fig. 5:**
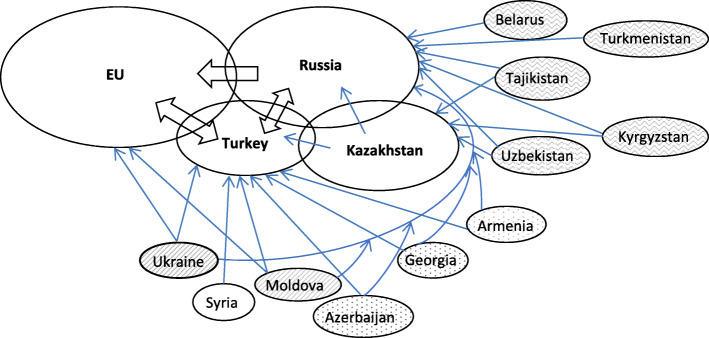
Migration order and migration systems in Europe, Eurasia and North Asia

In popular discourses the EU is considered the main destination in the region and globally, similar to North America, Australia and the Gulf countries. In section 3 it is shown that developments across some key domains of Russia and Turkey, notably the economy, demography, policy and infrastructures not only changed their position in the global economic and political orders but coincide with the migration transitions of both countries which subsequently turned them into major migration receiving countries. The impact is not limited to both countries itself; instead, these developments diversify global migration and fundamentally change the configuration of states with regards to global migration processes. There are now an increasing number of poles of attractions offering more choice to people aspiring or having to migrate. Migration desires, as depicted above, are an important indicator for these developments. This diverts and partly also eases migration to the EU. On the other hand, the EU may partly even need to compete with Russia, Turkey and other new immigration countries over attracting skilled and unskilled migrants (e.g. Shachar 2006; Bhagwati and Hanson 2009; Boeri et al. 2012). From an analysis of the migration transitions and new flows an intricate map of sending and receiving countries of migration emerges (Fig. 5). Many flows are dual- or even multi-directional, as from Uzbekistan to Russia and Kazakhstan or from Ukraine to Russia, the EU and Turkey. There are also reverse flows meaning that even major receiving countries, notably Russia, Kazakhstan and Turkey are also sending countries of migrants and that major sending countries such as Ukraine still also receive small numbers of immigrants. Four types of sending countries can be identified: (i) in Belarus, Turkmenistan, Tajikistan, Kyrgyzstan and Uzbekistan people mainly migrate to Russia and/or Kazakhstan; (ii) in Moldova and Ukraine people simultaneously migrate to Russia, the EU and Turkey; (iii) in Azerbaijan, Georgia and Armenia migrants mainly move to Russia and Turkey and (iv) in Syria migrants primarily move to Turkey and sometimes on to the EU or alternatively to Lebanon, Jordan and the EU but not Russia. To this we have to add Israel, Libya and Egypt as other major migrant receiving countries in the vicinity of Europe; even Morocco now also hosts some immigrants. China is another emerging player in all this and requires further research. The analysis finds that the sum of these bi- or trilateral processes or migration systems involve a large number of countries across various geographic regions (Fig. 5) and can be depicted as ‘structured, regular, logic and patterned migration processes’ (see section 3). These, I accordingly conceptualise as a dynamic multipolar migration order.

**Fig. 6:**
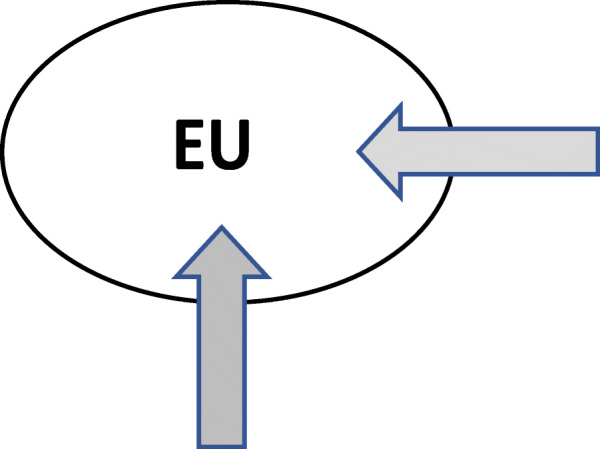
Depiction of Eurocentric migration discourse

**Fig. 7:**
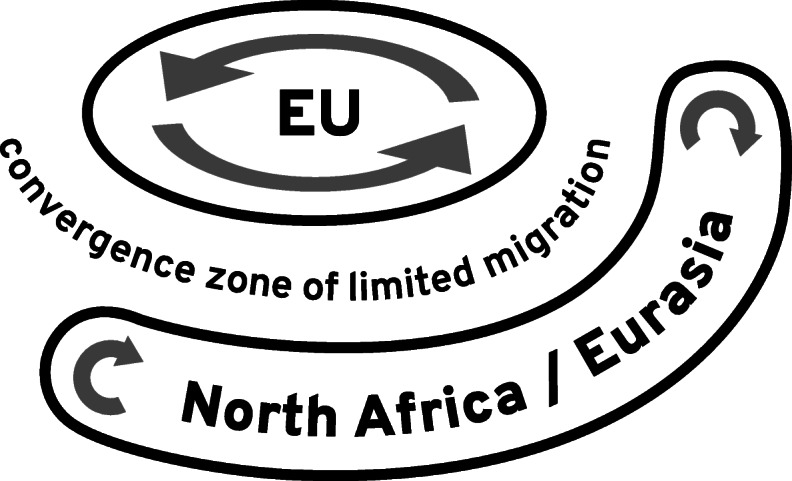
Depiction of actual migration situation

From Fig. 5, it occurs that a belt of further destination countries has been emerging around the EU that stretches from Morocco along the southern rim of the Mediterranean to Turkey and on to Russia and Kazakhstan and thus from Tangier via Cairo, Tel Aviv, Istanbul and Moscow to Vladivostok. Migration within the EU, notably the Schengen country zone, as well as within this belt is often visa-free, only migration across the two groups of countries is largely restricted. Hence, the old order with the EU as main destination region has been replaced by a new order of partly separated migration systems with several main receiving countries. Conceptualising the configuration of states in migration processes as a migration order, aligns, as suggested here, migration studies with studies of social transformation as well as the world economic and political order, enables seeing the bigger picture beyond national containers and suggests querying outdated discursive frames (see Figs. 6 and 7). This also fills a gap in migration studies in so far there is not yet a consolidated concept for the global structure of states in migration processes.

## Data Availability

All data generated or analysed are available from the sources quoted in this article.

## Footnotes

1 The impact of the coronavirus crisis is not taken into here

2 Whereas Merton (1968: 71) refers to ‘propositions about the structure of society and its changes, the behavior of man within that structure and the consequences of that behavior’ I basically rephrase this as ‘structure of states with regards to migration, the behaviour of man with that and the consequences of that behaviour’

3 Varying length of stay, only for varying purposes and sometimes only for specific types of persons

4 Armenia, Azerbaijan, Belarus, Kazakhstan, Kirgizstan, Moldova, Tajikistan, Ukraine and Uzbekistan, no restrictions; Bosnia and Herzegovina, Croatia, Macedonia, Montenegro and Serbia, time limits apply

5 Argentina, Brazil, Chile, Columbia, Cuba, Nicaragua, Peru, Venezuela, Hong Kong, Israel, Thailand and Turkey

6 Unfortunately, national and local data on flows and immigration to Turkey from before 2014 is imprecise and incomplete which requires a cumbersome aggregation of migration data from many different sources (Düvell, Soyuşen & Çorabatır 2015). The 2013 Law on Foreigners and International Protection changed this and generates better statistics

7 Most EU countries except UK, Ireland, Netherlands, Belgium, Malta, Croatia and Austria (they can obtain e-visa), Russia, Belarus, Ukraine, Georgia, Azerbaijan, Kirgizstan, Tajikistan, Kazakhstan, Uzbekistan, Moldova, Serbia, Pakistan, Japan, Hong Kong, Indonesia, Malaysia, Iran, Israel, Syria, Lebanon, Jordan, Qatar, Yemen, Algeria, Libya, Tunisia, Morocco, Brazil, Argentina, Columbia, Bolivia, Uruguay etc., e-visa are issued to travellers from Canada, Armenia, Saudi Arabia, Kuwait, India, Iraq, South Africa and others though both lists frequently change
